# Investigation of *Euphorbia nivulia*-HAM for Enzyme Inhibition Potential in Relation to the Phenolic and Flavonoid Contents and Radical Scavenging Activity

**DOI:** 10.3390/life12020321

**Published:** 2022-02-21

**Authors:** Muhammad Younus, Muhammad Mohtasheem-ul-Hasan, Shakeel Ijaz, Muhammad Kamran, Ambreen Maqsood, Bushra Saddique, Uzair Nisar, Muhammad Ashraf, Eman A. Mahmoud, Ahmed M. El-Sabrout, Hosam O. Elansary

**Affiliations:** 1 Department of Pharmacognosy, Faculty of Pharmacy, The Islamia University of Bahawalpur, Bahawalpur 63100, Pakistan; mkhan@iub.edu.pk; 2 Department of Pharmacognosy, Faculty of Pharmacy, University of Karachi, Sindh 75270, Pakistan; mohassan@uok.edu.pk; 3 School of Pharmacy and Pharmaceutical Sciences, Trinity College Dublin, The University of Dublin, D02 PN40 Dublin, Ireland; 4 Department of Pharmaceutics, Faculty of Pharmacy, The Islamia University of Bahawalpur, Bahawalpur 63100, Pakistan; 5 School of Agriculture, Food and Wine, The University of Adelaide, Adelaide 5005, Australia; 6 Faculty of Agriculture, The Islamia University of Bahawalpur, Bahawalpur 63100, Pakistan; ambreenagrarian@gmail.com (A.M.); bushra.siddique@iub.edu.pk (B.S.); 7 Department of Pharmacology, Faculty of Pharmacy, Ziauddin University, Karachi 75600, Pakistan; eagle.ka4u786@yahoo.com; 8 Department of Chemistry, The Islamia University of Bahawalpur, Bahawalpur 63100, Pakistan; dr.m.ashraf@gmail.com; 9 Department of Food Industries, Faculty of Agriculture, Damietta University, Damietta 34511, Egypt; emanmail2005@yahoo.com; 10 Department of Applied Entomology and Zoology, Faculty of Agriculture (EL-Shatby), Alexandria University, Alexandria 21545, Egypt; elsabroutahmed@alexu.edu.eg; 11 Plant Production Department, College of Food & Agriculture Sciences, King Saud University, Riyadh 11451, Saudi Arabia

**Keywords:** medicinal plant, oxidative damage, urease inhibition, α-glucosidase inhibition, acetylcholinestrase inhibition

## Abstract

*Euphorbia nivulia*-Ham (EN) is a neglected medicinal plant traditionally used for a number of pathologies, but it has not been explored scientifically. In the current study, its various fractions were assessed for their phenolic and flavonoid content, radical scavenging, as well as its enzyme inhibitory potential. The hydro-alcoholic crude extract (ENCr) was subjected to a fractionation scheme to obtain different fractions, namely n-hexane (ENHF), chloroform (ENCF), n-butanol (ENBF), and aqueous fraction (ENAF). The obtained results revealed that the highest phenolic and flavonoid content, maximum radical scavenging potential (91 ± 0.55%), urease inhibition (54.36 ± 1.47%), and α-glucosidase inhibition (97.84 ± 1.87%) were exhibited by ENCr, while the ENBF fraction exhibited the highest acetylcholinestrase inhibition (57.32 ± 0.43%). Contrary to these, hydro-alcoholic crude as well as the other fractions showed no significant butyrylcholinestrases (BChE) and carbonic anhydrase inhibition activity. Conclusively, it was found that EN possesses a significant radical scavenging and enzyme inhibitory potential. Thus, the study may be regarded a step forward towards evidence-based phyto-medicine.

## 1. Introduction

The genus *Euphorbia* comprises about 2000 species, with a number of medicinally active plants being used in folklore medicine in different parts of the world, since prehistoric times [1,2,3]. One of the members of the family Euphorbiacae, *Euphorbia nivulia* Buch.-Ham. (EN), has gained the attention of researchers due to its biological activities. There is not much literature available on the biological activities of *Euphorbia nivulia* [4]. It has also gained the attention of researchers for its pharmacological activities and its potential medicinal use. Phytochemical studies reveal that EN is rich in phenolic and flavonoid compounds. Other than these, other compounds like terpenes including triterpenes and diterpenes, cyanogenic glycosides, alkaloids, tannins, cerebrosides, glycerols, and steroids are also present [4]. Traditionally, this plant has been used to cure swelling, urinary retention, worm infections, ear and skin disorders, to cure bone fractures, as a bronchodilator in asthma and chronic cough [5], hemorrhoids, rheumatic pain, jaundice, hepatomegaly, and splenomegaly [6]. Scientific studies have revealed that it has many potent pharmacological activities like anticonvulsant [7], antibacterial, antifungal [8], hemostatic, wound healing, cytotoxic activities [9].

Northern and central India is the habitat of the plant, where it is planted as a hedge plant, often in dry areas, and is found wild in arid soils. The species is widely distributed in tropical Asia, Africa, Europe, and Australia, and is also found in India, Myanmar, and Pakistan [10]. Flowers are reddish with 1-cm long peduncles. The flowering and fruiting period is March to July [11,12]. Chemically, it contains tetracyclic trierpenes and ingol diterpenes [9]. Lectin, a high molecular weight glycoprotein [13], and *Nivulia-II* and *Nivulian-III*, two other glycoproteins, have been isolated from the latex [14]. The latex also contains phenolic compounds, alkaloids, cynogenic glycosides, terpenes, and tannins [15]. Miscellaneously, it contains citric, tartaric and mallic acids, eupol, nerifoiol, fat, albuminoids, hydrolytic enzymes, etc. Phytoelements like Fe (1.48), Cu (0.072), Zn (0.38), Mn (0.173), Mg (0.204), Na (2.08), and Ca (1.031) have been detected in ppm quantities by atomic absorption spectroscopy [16]. All parts of the plant possess medicinal properties, and mostly the juice or latex of different parts is used traditionally.

Nature has provided many hidden ways to cure different ailments, and enzyme inhibition is one of them. Inhibition of many important enzymes is a pivotal area of interest in pharmacological and pharmaceutical research, and is known to be involved in the discovery of new and potential therapeutic candidates. It is estimated that nearly 47% of the total available drugs work by inhibiting different enzymes as an essential target [17]. α-amylase, α-glucosidase, xanthine oxidase, acetylcholinesterase (AChE), butyrylcholinesterase (BChE), and carbonic anhydrase are examples of a few enzymes that are physiologically and pharmacologically very important [18]. Over production and over stimulation of these enzymes may be the only reason for certain serious ailments, e.g., hyperglycemia, neurodegeneration, neuro-motor disorders, urolithiasis, pyelonephritis, and blindness. Thus, the inhibition of certain important enzymes could be helpful to counteract many serious pathological disorders associated with over activity [19]. The plants possessing a strong ROS scavenging activity also possess an enzyme inhibition potential [3].

Acetylcholine is one of the important neurotransmitters present that carries out cholinergic neurotransmission [20]. It is involved in many functions of the brain, like memory and restoring the balance among other neurotransmitters within the brain, and it also regulates many cognitive functions. It is reported in many studies that an imbalance of these regulations opens a door for many serious neurodegenerative disorders such as Alzheimer and Parkinson disease [21]. Acetylcholine estrases (AChE) are the main resident of the excitable tissues in the CNS, whereas butyrylcholinesterases (BChE) are present in both the central and peripheral nervous system. These lead to acetylcholine degradation within the cholinergic synapse, resulting in neurodegenerations [22].

Enzyme inhibition may offer a potential basis for the discovery of new therapeutic agents [23,24]. Acetylcholinesterase (AChE), butyrylcholinesterase (BChE), α-glucosidase, urease, and carbonic anhydrase are examples of a few enzymes that are physiologically and pharmacologically very important. An over production and/or over stimulation of these enzymes is associated with certain serious ailments, e.g., hyperglycemia, neurodegeneration, neuro-motor disorders, peptic ulcers, urolithiases, pyelonephritis, and blindness. Thus, their inhibition could be helpful to counteract these serious pathological disorders [24]. Previous studies have shown that several members of the genus *Euphorbia* have shown inhibitory activities against a wide range of enzymes, and have proven their potential as a potent enzyme inhibitory therapeutic agent to be used in wide variety of diseases [25,26]. Because of these reasons, the present study was designed to assess phenolic, flavonoid content, in-vitro radical scavenging, and enzyme inhibitory potential of EN using different chemical and biochemical enzymes assays. Targeting these enzymes could be a correct approach for treating many disorders, including Alzheimer disease [27]; memory loss; epilepsy; diabetes; kidney stone formation; liver disorders, like hepatitis and liver cirrhosis; digestive tract disorders, like gas accumulation, dyspepsia, and indigestion; and ulcers. Regulating various enzyme functions may be instrumental in the prevention and treatment of cancers, and cardiac and glandular disorders, like the rapidly wide spreading diseases.

There are not much data available about this plant, thus it is lacking satisfactory scientific information. To the best of our knowledge, the current study is the first of its kind exploring the enzyme inhibition potential of the plant.

## 2. Results and Discussion

### 2.1. Extraction Yield

The extraction yields of crude and various fractions of EN were calculated and are presented in Table 1, which shows the percentage yield of ENCr, ENHF, ENCF, ENBF, and ENAF was 7.14, 2.5, 2, 15, and 84%, respectively.

### 2.2. Total Phenolic and Flavonoid Content Estimation

Phenolic and flavonoid compounds are very important phytochemicals of plants that have crucial role in their defense, owing to their free radical scavenging ability. The present results revealed that EN contains promising phenolic and flavonoid content, as shown in Table 1. The highest total phenolic content was exhibited by ENCr (127 ± 3.32 mg GAE/gE), followed by ENBF (123 ± 1.78 mg GAE/gE), ENCF (97 ± 1.45 mg GAE/gE), ENAF (77 ± 1.98 mg GAE/gE), and ENHF (66 ± 1.57 mg GAE/gE). Similarly, the highest flavonoid content was exhibited by ENCr (70 ± 2.96 mg QE/gE), whereas the lowest was in ENHF (38 ± 1.39 mg QE/gE). Previously described work revealed Euphorbia species to possess an abundance of phenolic and flavonoid compounds [28]. ENCr and its fractions exhibited a promising antioxidant potential because of natural antioxidants, especially flavonoids and phenolic acids, as these polyphenols prevent oxidation. Preliminary phytochemical screening of EN and fractions have shown that the plant contains phenolic acids and flavonoids, and HPLC analysis confirmed various phenolics and flavonoids in this species. Phenolics and flavonoids possess an antioxidant capacity, so these may prevent several degenerative diseases like ageing, diabetes, and cancer, and may protect the human body.

### 2.3. Radical Scavenging Potential Estimation

**Figure 1:**
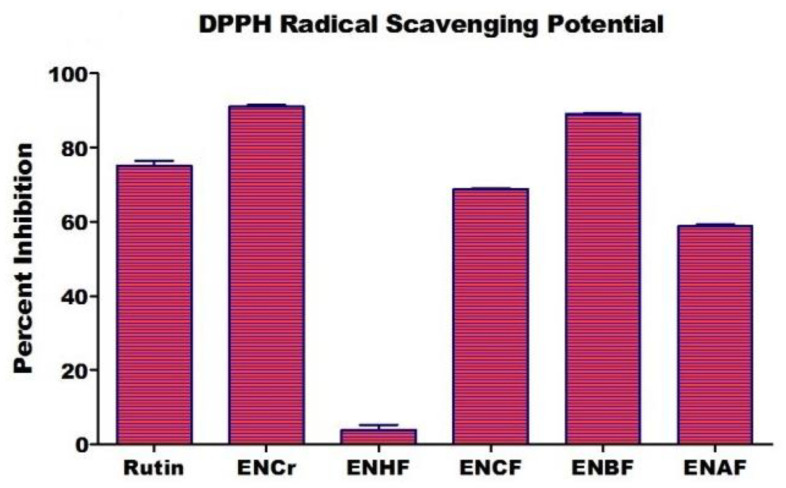
Enzyme inhibitory activity of various fractions of EN extract on the DPPH radical scavenging potential. All values are expressed as mean ± SD, where n = 3.

Previous studies have revealed that plants of *Euphorbia* species including EN possess a very promising radical scavenging potential. In the present study, at 0.5 mg/mL, the maximum radical scavenging potential was exhibited by ENCr (91 ± 0.55%), followed by ENBF (89 ± 0.43%), ENCF (68.80 ± 0.29%), ENAF (58.80 ± 0.52%), and ENHF (3.87 ± 1.43%), as indicated in Figure 1. These results are in accordance with previous studies on Euphorbia species, which showed that the polar fractions have more radical scavenging potential than non-polar ones [29]. The main finding in this study was that our selected medicinal plant species showed a stronger antioxidant activity, as it contained more phenolics. A direct relationship exists between antioxidant activity and total phenolic content, as phenolic compounds have a major contribution towards antioxidant activity. The antioxidant potential of phenolics is correlated with the total phenolic contents and chemical structures. The structure activity relationship of phenolics, their free radical scavenging, and their antioxidant property is dependent on the number and position of hydrogen-donating hydroxyl groups in the aromatic ring of phenolics. Oxidative damage caused by oxidation reactions can be decreased by using natural antioxidants. Antioxidants are employed in the treatment of many diseases by decreasing the adverse effects of free reactive species. An over-production of free radicals or ROS results in oxidative damage of macro-molecules, as well as a number of disorders including Atherosclerosis and Alzheimer disease, and inflammation, neurodegenerative, diabetes, liver, kidney, and cardiovascular diseases. ROS also plays a key role in the ageing process and certain types of cancers. Moreover, free radicals also cause food impairment, so it is important to stop the production of free radicals in foods and organisms.

#### 2.3.1. Acetylcholinesterase and Butyrylcholinesterase Inhibition Estimation

The plants possessing a strong ROS scavenging activity also possess an enzyme inhibition potential. Acetylcholinestrases (AChE) are the main resident of the excitable tissues of the body in the CNS, whereas butyrylcholinestrases (BChE) are present in both the central and peripheral nervous systems. They are involved in the acetylcholine breakdown at the cholinergic synapse, which leads to cognitive decline in Alzheimer disease. Their inhibition could be a curative approach towards these neurodegenerative disorders [30]. In the present study, crude and various fractions of EN showed a good AChE inhibitory potential when tested at a concentration of 0.5 mg/mL, as depicted in Table 2. Among these, the ENBF fraction exhibited highest (57.32 ± 0.43%) and the ENHF fraction showed lowest (35.61 ± 0.36%) inhibitory activity. The use of some members of the *Euphorbiaceae* family as antioxidants in association with inhibitors of acetylcholinesterase (AChE), the fundamental enzyme in the breakdown of acetylcholine, is considered as one of the most promising approaches for Alzheimer disease treatment. Previous data have shown that other Euphorbia species possess an AChE inhibitory activity [31]. ENE and various fractions revealed a high antioxidant and good acetylcholinesterase inhibitory activity due to their rich total polyphenolic and flavonoid contents. Contrary to this, the crude extract of EN and its fractions did not show a significant BChE inhibitory activity (Table 3). This is not surprising because there are distinct separate inhibitors and substrate specificities expressed by AChE and BChE [32].

#### 2.3.2. α-Glucosidase Inhibition Estimation

The *α*-glucosidase enzyme causes degradation of oligosaccharides (branched chain) and is categorized as a major source of hyperglycemia by releasing glucose [33]. The inhibition of this enzyme can be a potential approach for maintaining the glucose level within the optimized normal range. In the present study, EN exhibited an excellent α-glucosidase inhibitory activity, as shown in Table 4. The results revealed that the ENCr fraction presented a maximum enzyme inhibition (97.84 ± 1.83%), followed by ENBF (97.81 ± 1.87%), ENHF (97.42 ± 1.59%), ENCF (71.52 ± 1.62%), and ENAF (16.32 ± 1.38%) at 0.5 mg/mL. Surprisingly, this inhibitory activity was more than that the activity expressed by the standard used (92.68 ± 0.91%), and similar results have been observed with other Euphorbia species [34]. This might be attributed to the presence of phenolic and flavonoid compounds and the antioxidant activity of the EN extract [35]. Polyphenols, flavonoids, and other antioxidants have been reported to inhibit α-amylase and α-glucosidase. In the current study, ENE and fractions showed a significant inhibitory capacity towards the key enzyme, i.e., α-glucosidase, linked to metabolic ailments such as type II diabetes. The literature survey reveals that many medicinal plants with an inhibitory effect on the α-glucosidase enzyme are categorized as potential antidiabetic agents. It has also been reported in many studies that plants show α-glucosidase inhibition due to the presence of flavonoids as a chemical entity.

#### 2.3.3. Urease Inhibition Estimation

Urease is responsible for the catalysis of urea to ammonia, producing a neutralized environment favorable for the colonization and virulence of certain bacteria such as *H. Pylori* and *P. mirabilis*. These are involved in the pathogenesis of peptic ulcers and stomach cancers, as well as pyelonephritis, urolithiasis, and urinary catheter encrustation, etc. In the current study, ENCr manifested a maximum urease inhibition of 54.36 ± 1.47% at 0.5 mg/mL, as displayed in Table 5. All other fractions did not show a good inhibitory activity. These results are somewhat contrary to previous studies, in which natural urease inhibitors with a very good activity from Euphorbia species have been previously reported [36,37].

#### 2.3.4. Carbonic Anhydrase Inhibition Estimation

Carbonic anhydrase plays important roles in mammals, such as through gas balance, pH control, secretion of electrolytes and ion transport, calcification, and tumorigenesis [38]. In the present study, no carbonic anhydrase inhibitory activity was exhibited by crude or any fraction of EN when compared with Acetazolamide as the standard (Table 6). To date, no carbonic anhydrase activity by *Euphorbia* species has been found in the literature. However, contrary to these results, *Euphorbia hirta* exhibited aa diuretic activity similar to that of acetazolamide by excreting Na^+^, K^+^, and HCO_3_^−^ [39].

### 2.4. Chemicals and Equipment

All of the chemicals and reagents used in this experimental work were of analytical grade. Reagents Tris, eserine, acetylcholinesterase, butylated hydroxytoluene, acetylthiocholine iodide, HEPES, carbonic anhydrase, acetazolamide, 5, 5′-Dithiobis (2-nitrobenzoic acid) and 4-nitrophenyl acetate, Folin−Ciocalteu reagent, and ethanol were purchased from Sigma-Aldrich. Dimethyl sulfoxide was obtained from Merck (Darmstadt, Germany).

### 2.5. Plant Material

The plant material (aerial parts including leaves, shoots, stem, and flowers) was collected from adjoining areas of the Bahawalpur region, Pakistan. It was authenticated by an expert taxonomist from the Department of Botany, The Islamia University of Bahawalpur. A voucher specimen (No. EN-AP-05-12-041) was deposited in the herbarium of the Pharmacology Research Lab, Department of Pharmacy, The Islamia University of Bahawalpur, Pakistan, for future reference.

### 2.6. Extraction and Fractionation

Dried powdered plant material (aerial parts) (macerated in 70% aqueous ethanol) yielded a thick and semi-solid, dark brown gummy mass. Then, 10 kg of dried powdered plant material (aerial parts) was macerated in 70% aqueous ethanol at room temperature for 15 days using the cold maceration method with occasional stirring. Each time, 12 L of aqueous ethanol was used to soak the powder. The macerated mixture was filtered every three times with muslin cloth separately, and then further filtration was done by Whatman Grade-1 filter paper. The filtrate was then evaporated under reduced pressure (−760 mm Hg) and controlled temperature (at 45–50 °C) on the rotary evaporator. A thick and semi-solid, dark brown gummy mass was obtained, which was then placed in an oven. The dried material was weighed; the percentage yield was calculated and then stored at 4 °C in a refrigerator in an air tight container. Furthermore, successive solvent extraction was used to fractionate the aqueous ethanolic extract using various solvents of increasing polarity, including n-hexane, chloroform, n-butanol, and water, as previously described [40]. The fractions were also subjected to drying in an oven to increase the concentration under reduced pressure. These were dried, weighed, labeled, and then stored at 4 °C in a refrigerator in air tight containers. Aqueous ethanolic crude extract and the four fractions obtained in this way were named as follows: 

ENCr = 70% aqueous ethanolic crude extract; ENHF = n-hexane fraction; ENCF = chloroform fraction; ENBF = n-butanol fraction; ENAF = aqueous fraction.

### 2.7. Total Phenolic Content Estimation

The total phenolic content (TPC) of the EN crude extract and its various fractions was estimated according to a previously described method, with some modifications [41]. Briefly, an aliquot of 0.3 mL of sample (various concentrations) was mixed with 2.25 mL of Folin–Ciocalteu reagent. This reaction mixture, after being incubated at room temperature for 5 min, was added to 20 to 25 mL of Na_2_CO_3_ (6%). The resultant mixture was allowed to stand for 90 min for the completion of the reaction and the absorbance was measured at 725 nm. TPC was calculated using the standard calibration curve (0 to 200 mg/mL) and data were expressed as milligram of gallic acid equivalent per gram of dry weight extract (GAE/g). The results were expressed as mean ± SD, where n = 3.

### 2.8. Total Flavonoid Content Estimation

The total flavonoid content (TFC) of EN crude extract as well as its fractions was estimated using a modified colorimetric method described previously [41]. Briefly, 100 mL of sample solution (in methanol) was mixed with 25 mL of 1% NaNO_3_ solution and incubated at room temperature for 5 min. Then, 10 mL of 10% AlCl_3_ solution was added to it and it was again incubated for 5 min to complete the reaction. Afterwards, 35 mL of NaOH (4%) solution was added to this reaction mixture and it was diluted with 30 mL of methanol. Finally, the absorbance was measured at 510 nm. TFC was calculated using the standard calibration curve (0 to 200 mg/mL) and data were expressed as milligram of quercetin equivalent per gram of dry weight extract (QE/g). The results were expressed as mean ± SD, where n = 3.

### 2.9. Radical Scavenging Potential Estimation

The radical scavenging potential of the EN crude extract and its fractions was measured by a DPPH reagent according to a previously described method, with some modifications [42]. First, 90 µL mixtures of 10 µL sample solution dissolved in methanol (5 mg/mL) and 0.3 mM DPPH solution were incubated in the dark for 30 min at room temperature in a 96-well plate. The absorbance was measured at 517 nm using a 96-well microplate reader (Multiskan GO; ThermoFisher Scientific, Boston, MA, USA). The absorbance for the standard and blank was also measured. The percentage of total inhibition of DPPH radicals was measured using the following equation. The experiment was performed in triplicate and the results are expressed as mean ± SD.

$$Inhibition\;\left(\%\right)=\left[\frac{Abs.\;of\;control-Abs.\;of\;test}{Abs.\;of\;control}\;\right]\times 100$$

### 2.10. Acetylcholinesterase Inhibition Estimation

A solution of 100 µL of the reaction mixture containing 60 µL of buffer Na_2_HPO_4_ with pH 7.7 was prepared. To this, 10 µL (0.5 mM) test compound and 10 µL (0.005 unit well) enzyme were added and it was then pre-read at 405 nm. It was incubated for 10 min at 37 °C. The reaction was initiated through the addition of 10 µL of 0.5 mM acetylthiocholine iodide (substrate), followed by the addition of 10 µL DTNB (0.5 mM). Eserine (0.5 mM) was used as a positive control. Absorbance was measured at 405 nm on a Synergy HT (Multiskan GO; ThermoFisher Scientific, Boston, MA, USA) microplate reader [43]. Percentage inhibition of the enzyme was calculated using the following formula. The experiment was performed in triplicate and the results are expressed as mean ± SD.

$$Inhibition\;\left(\%\right)=\left[\frac{Abs.\;of\;control-Abs.\;of\;test}{Abs.\;of\;control}\;\right]\times 100$$

### 2.11. Butyrylcholinesterase Inhibition Estimation

The butyrylcholinesterase inhibition activity was performed according to the same method as reported for acetylcholinesterase [43].

### 2.12. α-Glucosidase Inhibition Estimation

α-glucosidase inhibition was carried out with slight changes, as described by Pierre’s protocol [44]. In detail, 100 µL of the reaction mixture, consisting of 70 µL (50 mM) phosphate buffer saline with pH 6.8, and 10 µL (0.5 mM) test compound was prepared. Then, 10 µL (0.057 units) enzyme was added to it. All these contents were mixed, pre-incubated for 10 min at 37 °C, and pre-read at 400 nm. The reaction started with the addition of 10 µL (0.5 mM) substrate (*p*-nitrophenyl glucopyranoside). Acarbose was used as the positive control. After 30 min of incubation at 37 °C, absorbance was measured at 400 nm using a Synergy HT (Multiskan GO; ThermoFisher Scientific, Boston, MA, USA) 96-well microplate reader. Yellow color absorbance was produced due to the formation of *p*-nitrophenol. The percentage inhibition of the enzyme was calculated using the following equation. The experiment was performed in triplicate and the results are expressed as mean ± SD.

$$Inhibition\;\left(\%\right)=\left[\frac{Abs.\;of\;control-Abs.\;of\;test}{Abs.\;of\;control}\;\right]\times 100$$

### 2.13. Urease Inhibition Estimation

For the estimation of the urease inhibition, 6 mL phosphate buffer pH 7.0 was prepared and added in 20 mL urease enzyme (Jack bean urease); it was dispended in each well of the 96-well plates. It was incubated for 10 min at 25 °C and 5 mL test compound (1 mM concentration) was added to it. This mixture was further incubated at room temperature and after that 15 mL of 20 mM urea was added. It was again incubated for 10 min at 25 °C. The urease activity was determined by measuring ammonia production using the indophenol method, as described by [45]. Freshly prepared 115 mL phenol hypochlorite reagent, by mixing 45 mL phenol reagent (1% *w*/*v* phenol and 0.005% *w*/*v* sodium nitroprusside) and 70 mL alkali reagent (0.5% *w*/*v* NaOH and 0.1% active chloride NaOCl), was added in each well. Thiourea was used as the standard inhibitor of the urease. After incubation at room temperature for 25 min, absorbance was measured on an ELISA reader using Gen 5 software at 630 nm, and the percentage inhibition was calculated using the following formula [46]. The experiment was performed in triplicate and the results are expressed as mean ± SD

$$Inhibition\;\left(\%\right)=\left[\frac{Abs.\;of\;control-Abs.\;of\;test}{Abs.\;of\;control}\;\right]\times 100$$

### 2.14. Carbonic Anhydrase Inhibition Estimation

A carbonic anhydrase inhibition assay was performed according to [47], with slight modifications. In this assay, the formation of a yellow color compound 4-nitrophenol was measured. It was formed by the hydrolysis of 4-nitrophenyl acetate. Then, 20 mM buffer (7.4 pH) containing Tris and HEPES was used in the assay. Each well contained 140 µL of buffer, 20 µL of the freshly prepared solution of enzyme (0.1 mg/mL of deionized water) of purified bovine erythrocyte CA-II, and 20 µL of the test sample. The test sample was incubated for 15 min at 25 °C and pre-reading was taken at 400 nm using a Synergy HT (Multiskan GO; ThermoFisher Scientific, Boston, MA, USA) 96-well microplate reader. The reaction started with the addition of 4-nitrophenyl acetate. Then, 4-Nitrophenyl acetate was added in 20 µL at a concentration of 0.7 mM, and was it was diluted in ethanol and incubated at the same conditions for 30 min and after the reading was taken at 400 nm. The experiment was performed in triplicate and the results are expressed as mean ± SD. Percent inhibition was measured by the following formula:

$$Inhibition\;\left(\%\right)=\left[\frac{Abs.\;of\;control-Abs.\;of\;test}{Abs.\;of\;control}\;\right]\times 100$$

## 3. Conclusions

The present study investigated the phenolic contents, radical scavenging potential, and the enzyme inhibitory properties of EN. To the best of our knowledge, this was the first ever enzyme inhibition study for EN, as this plant has not been explored scientifically for its pharmacological activities. Based on the obtained results, it can be concluded that it is rich in phenolic and flavonoid contents, with a significant radical scavenging and enzyme inhibitory potential that makes EN extremely interesting and a potential candidate for further investigations to find novel and efficient enzyme inhibitors. The plant may be an effective candidate in the treatment of various diseases that may be caused due to disturbances in enzyme activity in the body. EN may be used in diseases related to certain vital body systems, including central nervous system, liver, pancreas, kidney, gastro intestinal tract, as well as Alzheimer disease, dementia, epilepsy, diabetes, hepatitis, kidney stone formation, and digestive disorders. The extract(s) may be formulated into suitable dosages, formed by performing further studies, which may be used by the ultimate consumer through the effective consumption of locally available plant species.

## Figures and Tables

**Table 1:** Percentage yield of hydro-alcoholic crude extracts and its various fractions.

Fraction	Yield g (%)	TPC (mg GAE/g)	TFC (mg QE/g)
ENCr	40.0 (8)	127 ± 3.32 *	70 ± 2.96 *
ENHF	1.96 (0.392)	66 ± 1.57	38 ± 1.39
ENCF	0.94 (0.188)	97 ± 1.45	47 ± 1.65
ENBF	1.3 (0.26)	123 ± 1.78 *	64 ± 1.49
ENAF	0.78 (0.156)	77 ± 1.98	56 ± 1.28

ENCr = 70% aqueous ethanolic crude extract; ENHF = n-hexane fraction; ENCF = chloro-form fraction; ENBF = n-butanol fraction; ENAF = aqueous fraction. The results were expressed as mean ± SD, where n = 3. * specifies significance at *p* < 0.05. A higher % age yield of ENCr shows that the maximum chemical constituents were soluble in 70% aqueous ethanol, and after that in the hexane fraction and next in the butanol fraction. This shows that the majority of constituents were organic in nature.

**Table 2:** Enzyme inhibitory activity of various fractions of EN extract on the acetylcholinesterase assay (AChE).

Sample	Inhibition (%) at 0.5 mg/mL	IC_50_ (μg/mL)
ENCr	48.36 ± 0.25	-
ENHF	35.61 ± 0.36	-
ENCF	49.24 ± 0.29	-
ENBF	57.32 ± 0.43	354.17 ± 0.41 *
ENAF	52.19 ± 0.52	484.29 ± 0.45
Eserine	91.46 ± 1.25	0.19 ± 0.05

ENCr = 70% aqueous ethanolic crude extract; ENHF = n-hexane fraction; ENCF = chloroform fraction; ENBF = n-butanol fraction; ENAF = aqueous fraction. Mean ± SD were taken for each value and analyzed by one-way analysis of variance. * specifies significance at *p* < 0.05.

**Table 3:** Enzyme inhibitory activity of various fractions of EN extract on the butyrylcholinesterase assay (BChE).

Sample	Inhibition (%) at 0.5 mg/mL	IC_50_ (μg/mL)
ENCr	15.76 ± 0.29 *	-
ENHF	12.19 ± 0.17 *	-
ENCF	14.23 ± 0.23 *	-
ENBF	18.45 ± 0.27 *	-
ENAF	23.17 ± 0.35 *	-
Eserine	83.75 ± 1.16	0.62 ± 0.08

ENCr = 70% aqueous ethanolic crude extract; ENHF = n-hexane fraction; ENCF = chloroform fraction; ENBF = n-butanol fraction; ENAF = aqueous fraction. Mean ± SD were taken for each value and analyzed by one-way analysis of variance. * specifies significance at *p* < 0.05.

**Table 4:** Enzyme inhibitory activity of various fractions of EN extract on α-glucosidase assay.

Sample	Inhibition (%) at 0.5 mg/mL	IC_50_ (μg/mL)
ENCr	97.81 ± 1.87	22.83 ± 1.53 *
ENHF	97.42 ± 1.59	62.56 ± 1.34
ENCF	71.52 ± 1.62	328.57 ± 1.25
ENBF	97.84 ± 1.83	47.65 ± 0.87
ENAF	16.32 ± 1.38 *	-
Acarbose	92.68 ± 0.19	37.49 ± 0.17

ENCr = 70% aqueous ethanolic crude extract; ENHF = n-hexane fraction; ENCF = chloroform fraction; ENBF = n-butanol fraction; ENAF = aqueous fraction. Mean ± SD were taken for each value and analyzed by one-way analysis of variance. * specifies significance at *p* < 0.05.

**Table 5:** The enzyme inhibitory activity of various fractions of EN extract on the α-glucosidase assay.

Sample	Inhibition (%) at 0.5 mg/mL	IC_50_ (µg/mL)
ENCr	54.36 ± 1.47	472.75 ± 1.14 *
ENHF	31.74 ± 0.59	-
ENCF	16.12 ± 0.62 *	-
ENBF	17.35 ± 0.75 *	-
ENAF	15.35 ± 0.43 *	-
Thiourea	98.21 ± 0.18	21.25 ± 0.15

ENCr = 70% aqueous ethanolic crude extract; ENHF = n-hexane fraction; ENCF = chloroform fraction; ENBF = n-butanol fraction; ENAF = aqueous fraction. Mean ± SD were taken for each value and analyzed by one-way analysis of variance. * specifies significance at *p* < 0.05.

**Table 6:** Enzyme inhibitory activity of various fractions of EN extract on the α-glucosidase assay.

Sample	Inhibition (%) at 0.5 mg/mL	IC_50_ (μg/mL)
ENCr	18.57	-

Mean ENCr = 70% aqueous ethanolic crude extract. Mean ± SD were taken for each value and analyzed by one-way analysis of variance.

## Data Availability

All data are available in this manuscript.
